# In vivo compartmental analysis of leukocytes in mouse lungs

**DOI:** 10.1152/ajplung.00140.2015

**Published:** 2015-08-07

**Authors:** Brijesh V. Patel, Kate C. Tatham, Michael R. Wilson, Kieran P. O'Dea, Masao Takata

**Affiliations:** Section of Anaesthetics, Pain Medicine and Intensive Care, Faculty of Medicine, Imperial College London, Chelsea and Westminster Hospital, London, United Kingdom

**Keywords:** alveolus, lung interstitium, pulmonary capillary vasculature, flow cytometry, leukocyte trafficking

## Abstract

The lung has a unique structure consisting of three functionally different compartments (alveolar, interstitial, and vascular) situated in an extreme proximity. Current methods to localize lung leukocytes using bronchoalveolar lavage and/or lung perfusion have significant limitations for determination of location and phenotype of leukocytes. Here we present a novel method using in vivo antibody labeling to enable accurate compartmental localization/quantification and phenotyping of mouse lung leukocytes. Anesthetized C57BL/6 mice received combined in vivo intravenous and intratracheal labeling with fluorophore-conjugated anti-CD45 antibodies, and lung single-cell suspensions were analyzed by flow cytometry. The combined in vivo intravenous and intratracheal CD45 labeling enabled robust separation of the alveolar, interstitial, and vascular compartments of the lung. In naive mice, the alveolar compartment consisted predominantly of resident alveolar macrophages. The interstitial compartment, gated by events negative for both intratracheal and intravenous CD45 staining, showed two conventional dendritic cell populations, as well as a Ly6C^lo^ monocyte population. Expression levels of MHCII on these interstitial monocytes were much higher than on the vascular Ly6C^lo^ monocyte populations. In mice exposed to acid aspiration-induced lung injury, this protocol also clearly distinguished the three lung compartments showing the dynamic trafficking of neutrophils and exudative monocytes across the lung compartments during inflammation and resolution. This simple in vivo dual-labeling technique substantially increases the accuracy and depth of lung flow cytometric analysis, facilitates a more comprehensive examination of lung leukocyte pools, and enables the investigation of previously poorly defined “interstitial” leukocyte populations during models of inflammatory lung diseases.

resident alveolar and interstitial leukocytes (6, 43) as well as infiltrating blood-derived leukocytes (3, 37, 47) play pivotal roles in the lung during health and disease. However, current experimental techniques in animal models do not allow discrimination between the precise locations of these cells across different lung compartments, thereby significantly limiting our understanding of pathophysiology. For instance, blood leukocytes start to play fundamental roles in the evolution of inflammatory lung diseases when they are marginated/sequestered within the lung microvasculature (6, 26, 47) and subsequently show distinct intercellular interactions during transendothelial and transepithelial migration (15, 25, 38, 59). Because the majority of investigations do not separate alveolar, interstitial, vascular-marginated, and blood leukocyte populations, precise phenotype changes and functions of leukocytes as they migrate through each of the different lung compartments remain unknown.

Many investigations assume that lavage of the alveolar space or flushing of the pulmonary vasculature removes almost all alveolar and vascular cells, respectively. However, this is indeed not the case. Considerable portions of resident alveolar macrophages still remain within the lung even with multiple aggressive lung lavages using EDTA (5, 14). Similarly, flushing the vasculature of the lung was found to leave behind a substantive number of cells that are marginated and still attached to the endothelium (2, 56), making the differentiation between lung interstitial and intravascular spaces very difficult. In addition, vigorous lung perfusion has been shown to have a negative impact on the preservation of lung architecture (2). Hence, these protocols do not allow a reliable and accurate assessment of cell localization and number. More recently, imaging techniques such as intravital microscopy have been utilized to investigate interstitial leukocytes, but these techniques only allow the labeling of two or three cellular epitopes, limiting the examination of complex cellular phenotypes (8, 26). Furthermore, their application is limited to the subpleural areas, which may not be representative of the whole lung (8, 27, 52). Morphometric analysis (e.g., light and electron microscopy) remains subjective, semiquantitative, and time consuming.

The aim of this study is to develop and validate an in vivo antibody labeling methodology to enable the flow cytometric separation of leukocyte subsets into the various lung compartments. A number of previous studies have utilized intravenous staining protocols to define vascular vs. tissue leukocyte population in various organs (2, 4, 47), but such techniques do not allow clear identification of the interstitial compartment within the lungs. Here we utilize, for the first time, a simultaneous in vivo dual intratracheal and intravenous administration of anti-leukocyte antibodies to positively discriminate the alveolar and vascular cells and through negative gating define the interstitial compartment. This protocol allows the identification, quantification, and classification of leukocytes into three compartments of the mouse lung, in particular the elusive lung interstitium. This simple yet powerful experimental approach will provide researchers the opportunity to further explore biological and immunological mechanisms important not only in the diverse range of inflammatory lung diseases but also in the maintenance of lung homeostasis during health.

## MATERIALS AND METHODS

All protocols were approved by the Ethical Review Board of Imperial College London and carried out under the authority of the UK Home Office in accordance with the Animals (Scientific Procedures) Act 1986, UK. Wild-type C57BL/6 mice (Charles River, Margate, UK) aged 10–12 wk and weighing 25–30 g were used for all experiments. We first developed the dual in vivo labeling technique using uninjured mice and then tested whether this method could be used to chase leukocyte trafficking across the lung compartments in disease states by using mice exposed to acid-induced lung injury.

### 

#### In vivo antibody labeling.

Animals were anesthetized by intraperitoneal injection of xylazine (8 mg/kg) and ketamine (80 mg/kg) and underwent tracheostomy whilst spontaneously breathing on inspired O_2_ fraction (Fi_O_2__) 1.0. The right external jugular vein was cannulated and 2 μg of a phycoerythrin (PE)-conjugated anti-CD45 antibody (diluted in 100 μl sterile PBS) was administered, together with heparin (10 IU/g), for systemic anticoagulation. After 5 min, allowing for the circulation of the antibody, animals were exsanguinated. The thoracic cavity was exposed through a midline sternotomy and the lungs were gently teased from their pleural adhesions. Subsequently, 5-0 silk sutures were placed carefully around the right and left main bronchi. One or both lungs underwent instillation (through the tracheostomy) with 0.5 or 1 ml sterile PBS, respectively, containing 2 μg/ml of a PE-Cy7 anti-CD45 antibody. Each suture was securely tied at the hilum to avoid spillage of the intratracheal antibody, and lungs were removed from the thoracic cavity. These ligated lungs were exposed to the intratracheal antibody for 5 min, after which they underwent processing for preparation of single-cell suspension.

#### Preparation of single-cell suspension.

Lungs underwent mechanical disruption by a GentleMacs tissue dissociator (Miltenyi Biotec) for 1 min in 2 ml of intracellular fixative (eBioscience). This technique allows immediate fixation and dissociation, enabling the best stability of epitopes (39, 43). The fixation reaction was stopped through the addition of 20 ml of ice-cold flow cytometry wash buffer (FWB)-PBS with 2% FCS, 0.1% sodium azide, and 5 mM EDTA. The suspension was subsequently sieved through a 40-μm nylon filter and washed again with 20 ml ice-cold FWB. The lung cell suspension was centrifuged at 4°C at 2,000 rpm. The cell pellet was resuspended in 1 ml of FWB and placed on ice.

#### In vitro antibody staining.

This was performed by incubation of lung single-cell suspension (or whole blood for reference of cell identification) with fluorochrome-conjugated antibodies for 30 min in the dark at room temperature. The following panel of antibodies for myeloid cell markers was used: CD45 (30-F11; BioLegend); CD11c (N418, eBioscience); CD11b (M1/70; BD Biosciences); F4/80 (CI:A3-1; BioLegend); CD103 (2E7; BioLegend); MHCII (M5/114.15.2; BioLegend); Ly6G (RB6-8C5; BioLegend); Ly6C (AL-21, Becton Dickinson Biosciences). A CD4 antibody (GK1.5, BioLegend) was used for T cell identification. After incubation 4 ml of FWB was added to lung cell suspension or 4 ml BD LyseFix solution to whole blood and samples were centrifuged at 2,000 rpm at 4°C for 5 min. Cells were resuspended in FWB and analyzed with a seven-channel CyAn ADP flow cytometer (Beckman Coulter). Prior to analysis the addition of AccuCheck counting beads (Invitrogen) enabled cell counts to be calculated. Data were analyzed with FlowJo software (Treestar).

#### Isolated perfused lung.

The efficacy of lung perfusion for the recovery of vascular leukocytes was evaluated by using the mouse isolated perfused lung (IPL) model previously established in our laboratory (18, 40, 56). In brief, anesthetized animals were injected with intravenous anti-CD45 antibody (with heparin to prevent intrapulmonary coagulation), exsanguinated, and placed on the IPL apparatus (Isolated Perfused Lung Size 1 Type 839; Hugo-Sachs Elektronik, March-Hugstetten, Germany). After tracheostomy and thoracotomy, the pulmonary artery and left atrium were cannulated, and lungs were ventilated (tidal volume 7 ml/kg, positive end-expiratory pressure 5 cmH_2_O, respiratory rate 80/min, with 21% O_2_ and 5% CO_2_ in N_2_) and perfused by RPMI 1640 without phenol red with 4% BSA in a nonrecirculating manner, at a rate of 25 ml·kg^−1^·min^−1^ with an left atrial pressure of 2.5 mmHg. Experiments were terminated at 1, 15, or 60 min, washed-out cells during this perfusion period were collected from the total IPL perfusate, and lung single-cell suspensions were also prepared from the postperfusion lungs. Both samples were analyzed by flow cytometry, and the numbers of vascular neutrophils labeled with in vivo-administered anti-CD45 antibody were quantified.

#### Acid-induced lung injury.

The in vivo labeling procedure underwent testing in our recently published mouse model of acid-induced lung injury (44). In brief, anesthetized animals were vertically suspended on a custom-made mount from their incisors by using a 2-0 suture. A nonthermal light source was used to transilluminate the trachea. Careful retraction of the tongue and laryngoscopy with blunt curved forceps allowed a grade 1–2 view of the vocal cords. A fine catheter was subsequently guided 1 cm below the vocal cords and 75 μl of an isoosmolar 0.1 M hydrochloric acid (pH 1.0) solution was instilled. Animals were left suspended for 1 min and then placed in a custom-made mouse high-dependency recovery area. During this recovery period they were actively warmed and given humidified supplemental oxygen, with Fi_O_2__ initially at 1.0 and then gradually reduced to 0.21 over 4 h. To ensure even distribution of the instillate, mice were rotated every 5 min until spontaneously moving. After this recovery period they were placed in isolated ventilated cages with air and free access to food and water for up to 4 days.

#### Statistical analysis.

Data are expressed as means ± SD. The model assumption of normality of residuals was assessed by QQ plot and the Shapiro-Wilk test. Statistical analyses of data were made by either a two-tailed Student *t*-test or one-way ANOVA with Bonferroni tests for intracompartmental comparisons between multiple time points (if parametric) and Mann-Whitney/Kruskal-Wallis tests (if nonparametric). Analyses of normal distribution were performed with SPSS version 20 (IBM) and statistical comparisons used GraphPad Prism (version 6.0f). A *P* value of less than 0.05 was considered significant.

## RESULTS

### 

#### Validation of compartmental staining protocol.

**Fig. 1.**
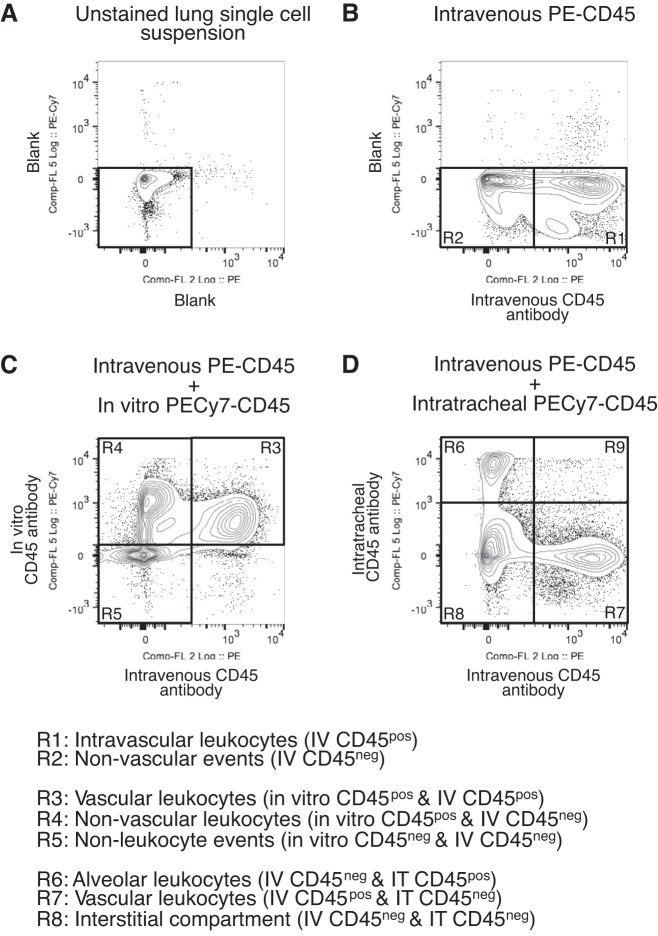
*A*: absence of antibodies shows a clear negative population of cells when lung single-cell suspension is analyzed with the phycoerythrin (PE) (*x*-axis) and PE-Cy7 (*y*-axis) channels. *B*: the in vivo intravenous (IV) injection of a PE-conjugated CD45 antibody leads to a clear shift rightward of an intravascular population (R1), leaving a nonvascular region (R2). *C*: subsequent labeling of whole lung single-cell suspension in vitro with a PE-Cy7 conjugated CD45 antibody leads to an upward shift of all leukocyte populations. Of note, all intravenously CD45-labeled leukocytes also stain positive for exogenous CD45 antibody (R3). Additionally, a population of cells emerges from the intravascular CD45-negative group that also express CD45 (R4). These are likely to be lung alveolar and interstitial leukocyte populations. *D*: when the same PE-Cy7-conjugated CD45 antibody is instilled intratracheally (IT), 1 population is labeled strongly positive (R6) and is likely to contain alveolar macrophages. The vascular compartment (R7) remains negative for the intratracheal antibody. The events that are dual negative for both intravenous and intratracheal CD45 antibodies is likely to contain interstitial leukocyte populations (R8). The number of events found in R9, representing the extent of bidirectional leak of antibody, is less than 0.5% of the total population. This confirms that the in vivo labeling enables separation of the alveolar, interstitial, and vascular compartments.

To investigate the compartmental localization of leukocytes, lungs were labeled “in vivo” through administration of an intravenous (PE-conjugated) followed by intratracheal (PE-Cy7 conjugated) anti-CD45 antibody in a step-by-step fashion (Fig. 1). Firstly, in the absence of intravenous and intratracheal labeling of the lung, events (after exclusion of debris using low forward scatter) of lung single-cell suspensions analyzed by flow cytometry are all negative in the PE and PE-Cy7 channels (Fig. 1*A*). The in vivo intravenous administration of a PE-conjugated anti-CD45 antibody produces a clearly separate population of CD45-positive cells (Fig. 1*B*, R1), which we define as cells within the “vascular” compartment, leaving a population of unlabeled nonvascular events (Fig. 1*B*, R2). Subsequently, this lung cell suspension (from animals given intravenous PE-conjugated CD45 antibody) was incubated in vitro with a PE-Cy7-conjugated CD45 antibody (Fig. 1*C*). As expected, the vascular population (R1) also stained positive for in vitro CD45 (R3). This also led to a population of CD45-positive cells (R4) emerging from the nonvascular gate (R2), leaving behind an unstained nonleukocyte CD45 negative population (R5). Finally, in vivo instillation (instead of in vitro incubation) of the PE-Cy7 conjugated CD45 antibody into the airways, following in vivo intravenous PE-conjugated antibody, stained a population of cells positive for intratracheal CD45 and negative for the intravenous CD45 (Fig. 1*D*, R6), which are highly likely to be resident alveolar macrophages. The intravascular population remains clearly defined (Fig. 1*D*, R7). The populations that are dual “negative” and not accessible to either the intratracheal or intravenous administered CD45 antibodies (Fig. 1*D*, R8) are likely to contain the interstitial leukocytes (as well as all other lung parenchymal cells). Importantly, there were virtually no dual-positive events (Fig. 1*D*, R9), suggesting minimal bidirectional leak of in vivo antibody staining between compartments. Importantly, the use of biexponential scaling (22, 42) provides a clear and distinct separation between alveolar (R6), vascular (R7), and interstitial (R8) populations.

#### Distinguishing between lung alveolar and interstitial compartments.

**Fig. 2.**
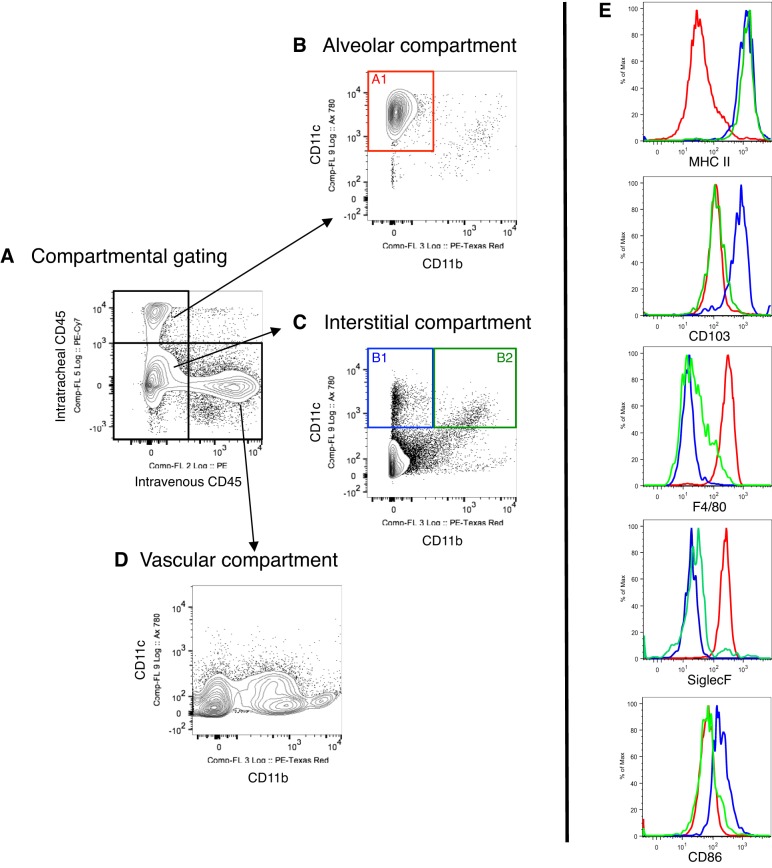
Distinguishing between the alveolar and interstitial compartments of the lung. *A*: in vivo labeling enables separation of the alveolar and vascular compartments from the rest of the lung homogenate. The interstitial compartment is dual negative to both intravenous and intratracheal CD45 antibodies. *B*: gating on the alveolar compartment shows that it predominantly contains a resident alveolar macrophage population (A1: red gate), which are relatively MHCII^neg^, CD103^neg^, F4/80^pos^, and SiglecF^pos^. *C*: the interstitial compartment [defined by those events not labeled by the intratracheal (PE-Cy7 CD45) and intravenous (PE CD45) antibodies] contains 2 populations of dendritic cells that are CD11c^pos^ and MHCII^pos^. One population is the intraepithelial-type conventional dendritic cells (cDC), CD11b^neg^CD103^pos^ (B1: blue gate), and the second is those cDCs resident in the lamina propria, CD11b^pos^CD103^neg^ (B2: green gate). *D*: the vascular compartment does not contain CD11c^pos^ events and only contains expected CD11b^pos^ events, which are described in subsequent panels. *E*: relative expression of cell surface markers in resident alveolar macrophages (red line); intraepithelial type cDC (blue line); and lamina propria-type cDC (green line) by compartmental analysis.

Analysis of the alveolar (PE-Cy7 CD45^pos^) compartment (i.e., intratracheal antibody positive and intravenous antibody negative) revealed that a CD11c^pos^CD11b^neg^ population accounted for over 95% of total alveolar events (Fig. 2*B*, gate A1). This population was also highly autofluorescent, MHCII^neg^ and CD103^neg^,and F4/80^pos^ and SiglecF^pos^, identifying them as resident alveolar macrophages, consistent with previous literature (6, 23, 32, 33). The remaining 5% of leukocytes within the alveolar compartment consisted of CD11c^pos^CD11b^neg^MHCII^pos^CD103^pos^ (2%) and CD11c^pos^CD11b^pos^MHCII^pos^CD103^neg^ (2%) cells, which are likely to be dendritic cell populations as described below, and CD11b^pos^Ly6G^pos^ neutrophils (1%).

Within the interstitial compartment (i.e., double negative for intratracheal PE-Cy7 and intravenous PE antibodies), there were two CD11c^pos^ leukocyte populations that can be clearly distinguished by CD11b expression (Fig. 2*C*, B1 and B2). These are both conventional dendritic cell (DC; cDC) populations since they have very high expression of MHCII. The two cDC populations can be further characterized by differences in CD103 expression and this has previously been shown to determine their specific location within the lung, intraepithelial vs. lamina propria (28, 55). Population B1 is CD11b^neg^CD103^pos^ (intraepithelial cDC) whereas population B2 is CD11b^pos^CD103^neg^ (lamina propria-located cDC). Furthermore, in contrast to resident alveolar macrophages, these DC populations have low or negligible F4/80 and SiglecF expression. The intraepithelial type cDC also shows slightly greater CD86 expression. In summary, the intratracheal labeling strategy enables us to clearly separate and distinguish between “alveolar” resident macrophages and “interstitial” resident dendritic cell populations. Table 1 describes an overview of basic phenotypic characteristics of myeloid cells within the three compartments of the mouse lung.

**Table 1.** Phenotypic characteristics of myeloid cell populations in the 3 compartments of the naive mouse lung

Cell Type	CD11c	CD11b	MHCII	CD103	F4/80	SiglecF	Ly6C	Ly6G	CD86	AF
*Alveolar compartment*										
Resident alveolar macrophage	+++	−	−	−	+++	+++	+++	−	+	+++
*Interstitial compartment*										
Intraepithelial cDC	+++	−	+++	+++	−	−	−	−	++	−
Lamina propria cDC	+++	+++	+++	−	+	−	−	−	+	−
Interstitial monocyte	−	++	+++	−	+	−	−	−	+++	−
*Vascular compartment*										
Classical "inflammatory" monocyte	−	++	+/−	−	+	−	+++	−	?	−
Nonclassical "resident" monocyte	−	++	−	−	+	−	−	−	−	−
Eosinophil	−	++	−	−	+	?	++	+	?	+++
Neutrophil	−	++	−	−	−	−	++	+++	?	+

+**++/++/+,** High/intermediate/low expression; −, no expression; ?, not tested; AF, autofluorescence; cDC, conventional dendritic cell.

#### Distinguishing between lung interstitial and vascular compartments.

**Fig. 3.**
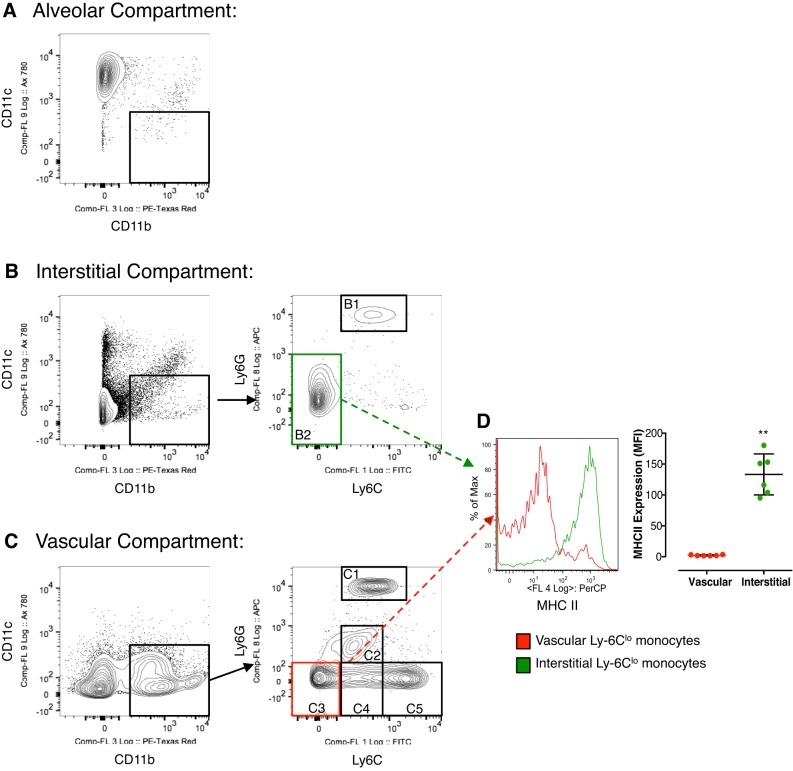
Distinguishing between the interstitial and vascular compartments of the lung. *A*: there are no CD11c^neg^CD11b^pos^ events in the alveolar space. *B*: the interstitium contains a CD11c^neg^CD11b^pos^ population that consists of some Ly6G^hi^ neutrophils (B1, <5%) but mainly Ly6C^lo^ monocytes (B2, 95%). *C*: the vascular space consists of a much larger population of CD11c^neg^CD11b^pos^ events. These are separated into neutrophils (C1, 35%), eosinophils [also Ly6G^inter^Ly6C^inter^ (C2), <5%], and monocytes [Ly6C^lo^ (C3), 25%, Ly6C^inter^ (C4), 10%, and Ly6C^hi^ (C5), 25%]. *D*: the interstitial Ly6C^lo^ monocytes (B2, green gate) have a significantly higher MHCII expression compared with the vascular Ly6C^lo^ monocyte (V3, red gate) population. This supports our notion that the compartmental strategy robustly distinguishes between interstitial and vascular compartments of the mouse lung. Data are means ± SD with *N* = 6; **P < 0.01 vs. vascular subset.

The in vivo labeling technique showed that virtually all CD11c^neg^CD11b^pos^ events are situated within the interstitial or vascular compartments, with minimal CD11c^neg^CD11b^pos^ events (<1% of alveolar events) within the alveolar space in healthy uninjured mouse lungs (Fig. 3*A*). The CD11c^neg^CD11b^pos^ leukocytes, within the interstitial compartment (Fig. 3*B*), consisted of a predominant population of Ly6C^lo^ monocytes (CD11c^neg^CD11b^pos^Ly6G^lo^Ly6C^lo^ cells, >95%; B2) with the suggestion of a minor neutrophil presence (CD11c^neg^CD11b^pos^Ly6G^hi^Ly6C^hi^ cells, less than 5%; B1). In contrast, the vascular compartment (Fig. 3*C*) showed greater proportion of neutrophils (C1; 35%) as well as a more varied proportion of monocyte subsets: Ly6C^lo^ (C3), 25%; Ly6C^inter^ (C4), 10%; and Ly6C^hi^ (C5), 25%. Given the limited number of flow cytometry channels, a complete exclusion of NK cells and eosinophils was not possible, but eosinophils (occupying ∼5% of events) are likely to be represented in gate C2 (CD11c^neg^CD11b^pos^Ly6G^inter^Ly6C^inter^ cells) (48).

Finally, interstitial Ly6C^lo^ monocyte populations were found to have a significantly higher expression of MHCII compared with their vascular Ly6C^lo^ monocyte counterparts (Fig. 3*D*). This clear separation in MHCII phenotype between interstitial and vascular Ly6C^lo^ monocytes confirms minimal leak of intravenous antibody into the interstitial space in the uninjured lung, further supporting the case that this in vivo labeling protocol allowed robust differentiation between cells within the vascular and interstitial compartments of the lung.

#### Compartmental analysis increases accuracy of anatomical location.

**Fig. 4.**
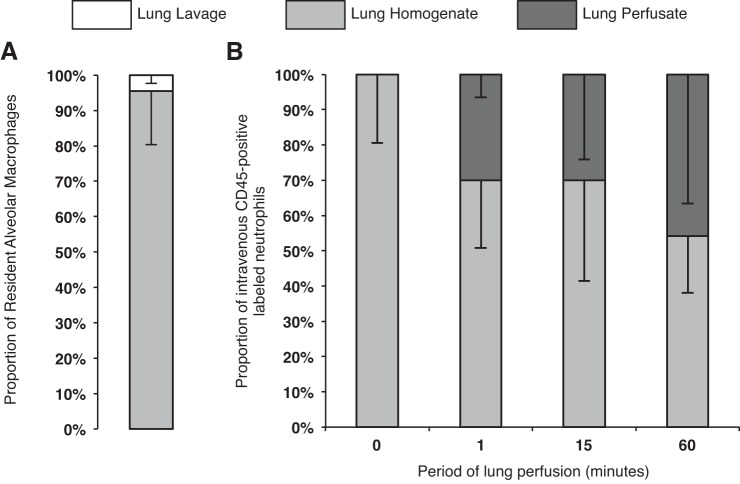
*A*: proportions of resident alveolar macrophages retrieved after 5 repetitive lung lavages by use of 1 syringe containing 750 μl normal saline (*N* = 4). *B*: proportions of neutrophils (labeled in vivo with intravenous anti-CD45 antibody) that are present in the lung perfusate and remaining postperfusion lung homogenate after varying periods of lung vascular perfusion (*N* = 4–6).

Current methods to analyze the anatomical location of leukocytes rely on both lung lavage (to remove alveolar cells) and pulmonary vascular perfusion (to remove vascular cells) with subsequent analysis of the lung homogenate (for interstitial cells) (5, 51). Thus a separate set of experiments was performed to assess the efficacy of lung lavage for retrieval of alveolar macrophages (Fig. 4*A*). Uninjured mouse lungs underwent five repetitive lavages via the endotracheal tube using one syringe containing 750 μl normal saline. The recovered lavage fluid and lung single-cell suspensions prepared from the remaining postlavage lungs were analyzed by flow cytometry. The numbers of resident alveolar macrophages (CD45^pos^CD11c^pos^CD11b^neg^F4/80^pos^) were 4.5 ± 2.4 × 10^4^ in the recovered lavage fluid vs. 9.4 ± 1.5 × 10^5^ in the lung homogenate. Hence, the overall retrieval of resident alveolar macrophages by this standard lavage procedure was found to be only less than 5%.

In another series of experiments, the efficacy of lung vascular perfusion for recovery of vascular leukocytes was evaluated by use of the IPL model. Mice underwent injection of intravenous anti-CD45 antibody for in vivo labeling of vascular leukocytes and subsequently underwent lung perfusion for 1, 15, and 60 min. The numbers of intravenously labeled CD45-positive neutrophils were counted in the washed-out cell populations in the IPL perfusate as well as in the remaining lung single-cell suspensions prepared from the postperfusion lungs (Fig. 4*B*). The results showed that up to 50% of vascular neutrophils labeled with the intravenous CD45 antibody still remain within the lung tissue even after 60 min of lung perfusion (Fig. 4*B*).

#### Application of compartmental analysis to experimental acid-induced lung injury.

**Fig. 5.**
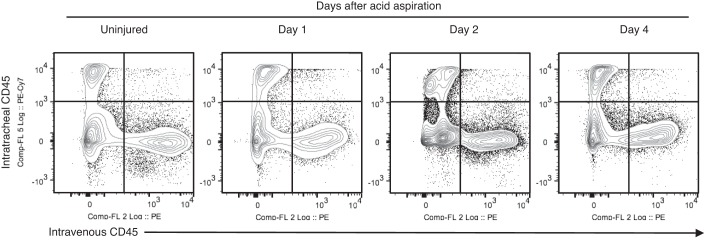
Changes in intravenous and intratracheal labeling during acid-induced lung injury. The compartmental approach maintains a clear separation between the 3 lung compartments despite significant barrier disruption during *days 1* and *2* of injury. The extent of bidirectional leak of in vivo applied antibody, as determined by dual-positive staining, remains unchanged (and less than 0.5% of all events) during the course of injury.

Analyses of the flux of leukocytes through the various compartments of lungs at various stages of lung injury are crucial to gain better understanding into pathophysiology. The methods described above were applied to our recently published acid aspiration model to gain a preliminary insight into the effectiveness of the compartmental strategy during injury. Animals underwent in vivo labeling at *days 1*, *2*, and *4* after acid aspiration, with *day 0* representing uninjured animals. These time points were predicted to best show influx and efflux/loss of leukocytes during inflammation and its resolution during lung injury (44). Figure 5 shows representative flow cytometry plots for the compartmental gating strategy as discussed previously in the uninjured mouse. The proportions of dual-positive events (Fig. 5), i.e., PE^pos^PE-Cy7^pos^, representing the extent of bidirectional leak of antibodies across the alveolar capillary barrier, remains unchanged (at <0.5%) between uninjured and injured animals even during significant alveolar edema at *days 1* and *2*. Indeed, we have previously shown that this acid-induced model produces a greater than 60% increase in lung water content with significantly increased lavage protein concentrations at *days 1* and *2* (44). Hence, this protocol allows consistent separation and gating of the three compartments in a model with severe disruption of the alveolar-capillary barrier.

**Fig. 6.**
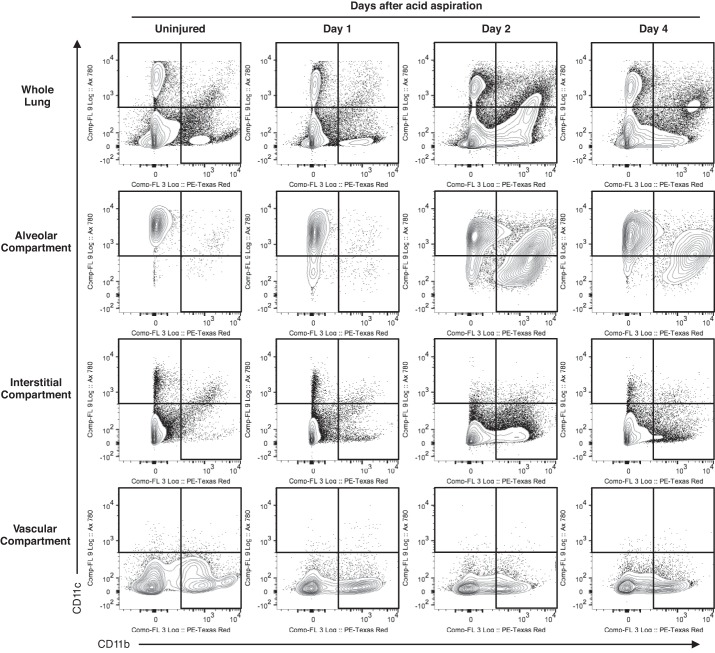
Compartmental gating showing changes in CD11b and CD11c events during injury (between *days 0* and *2*) and resolution (between *days 2* and *4*) of acid aspiration. There are increases in CD11b-positive events at *day 2* within the alveolar and interstitial spaces. As injury progresses, resident CD11c^pos^CD11b^neg^ alveolar macrophages in the alveolar compartment gain CD11b expression whereas recruited CD11c^neg^CD11b^pos^ cells gain CD11c.

Figure 6 shows the CD11c and CD11b characteristics of leukocytes after compartmental gating of the lungs as injury progresses. In the uninjured mouse lung, there are minimal numbers of alveolar CD11b^pos^ events and vascular CD11c^pos^ events. The alveolar compartment contains alveolar macrophages and some DC populations with no monocytes and negligible numbers of neutrophils. The interstitial compartment contains the two cDC populations and the CD11c^neg^CD11b^pos^Ly6C^lo^MHCII^pos^ subset of interstitial monocytes (as in Fig. 2). Immediately after acid aspiration, small numbers of CD11b^pos^ events are seen to enter the alveolar space as early as 3 h (not shown) and at *day 1*. However, the predominant CD11b^pos^ infiltration into the alveolar and interstitial compartments occurs on *day 2*.

**Fig. 7.**
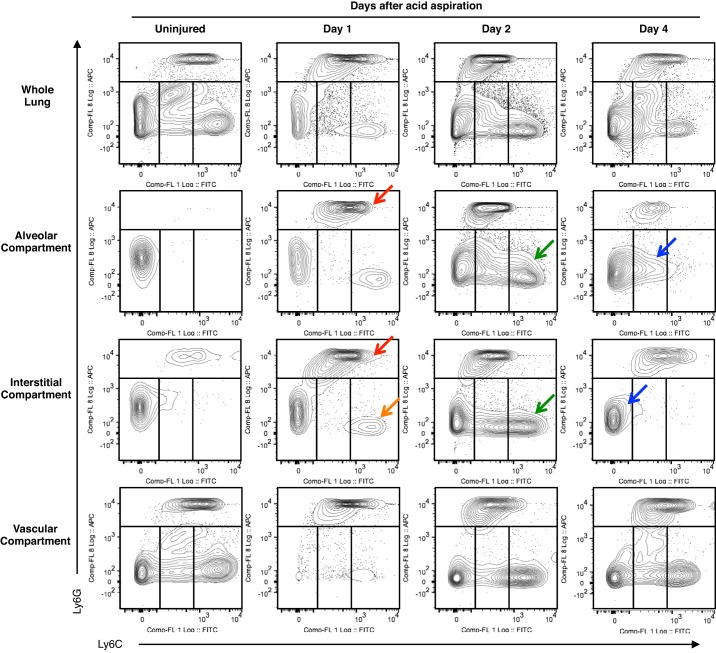
Changes to CD11b^pos^ subpopulations within each compartment during acid-induced lung injury. The alveolar and interstitial compartments show an increase in Ly6G^hi^ neutrophils on *day 1* (red arrows) as well as the beginnings of an infiltration of Ly6C^hi^ monocytes into the interstitial space (orange arrow). This is followed by a marked increase in Ly6C^hi/inter^ monocyte-derived exudative macrophages populations within the interstitial and alveolar compartment on *day 2* (green arrow). This population changes to a Ly6C^lo/inter^ phenotype on *day 4* (blue arrow). There are associated reductions in neutrophils within the alveolar and interstitial space consistent with the resolution phase of lung injury. The vascular compartment shows a reduction in monocyte numbers on *day 1*, which normalizes on *day 4*.

**Fig. 8.**
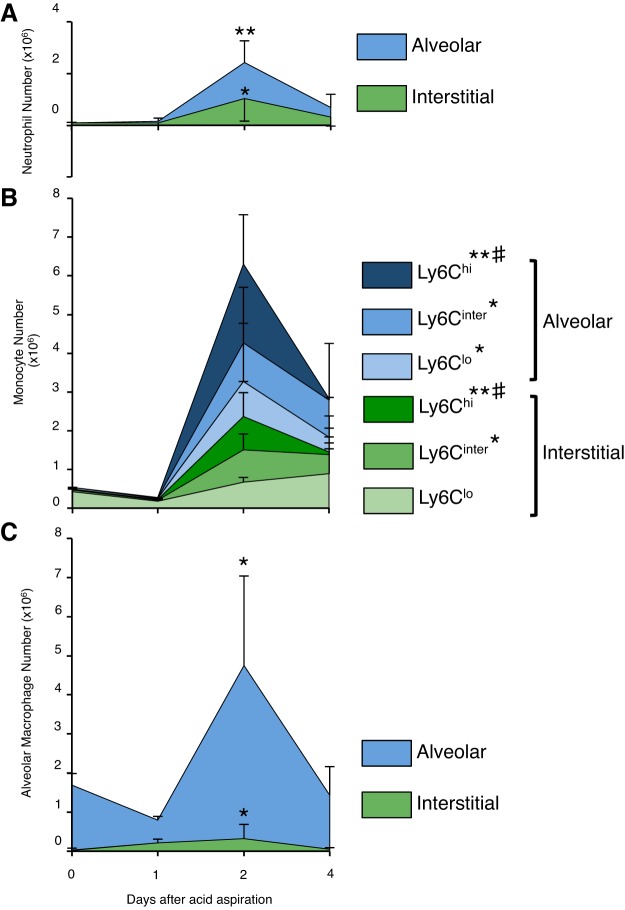
*A*: neutrophil numbers significantly increase on *day 2* after acid aspiration. Nearly 50% of all neutrophils are found within the interstitial space at this time point. Interstitial neutrophils may have been newly recruited. *B*: there is also a significant increase in monocyte migration into the interstitial and alveolar spaces on *day 2*. On *day 4*, whereas the numbers of Ly6C^lo/inter^ monocytes are maintained within the compartments, the numbers of Ly6C^hi^ monocytes reduce significantly in the alveolar compartment. *C*: there is a nonsignificant reduction in resident alveolar macrophage (CD11c^pos^CD11b^neg^F4/80^pos^) numbers on *day 1* of injury followed by a 3-fold increase in cells (on *day 2*) that have adopted a similar phenotype to resident alveolar macrophages. The replenishment of CD11c^pos^CD11b^neg^F4/80^pos^ cells coincides with the infiltration of exudative monocytes on *day 2*. The vascular space data have been omitted because this may be subject to variability as a result of differences in exsanguination between each animal. Data are means ± SD with *N* = 3–5/time point; *day 2* vs. *day 0*: *P < 0.05 **P < 0.01; *day 4* vs. *day 2*: #*P* < 0.05 (analysis performed within compartments).

Figure 7 shows a more focused analysis of CD11b^pos^ subpopulations within each compartment with Ly6G and Ly6C staining, while Fig. 8 shows a quantification of the dynamics of neutrophil and monocyte influx and efflux within the alveolar and interstitial lung compartments. There is an increase of Ly6G^pos^ neutrophil populations at 24 h of acid-induced lung injury in both alveolar and interstitial compartments. This neutrophil infiltration peaks at *day 2*, and at the same time we also see a large infiltration of CD11c^neg^CD11b^pos^Ly6G^lo^ monocyte-like cells, with a phenotype consistent with so-called “monocyte-derived exudative macrophages” (19), which we term “mono-mac” (Fig. 8, *A* and *B*). At *day 2*, this mono-mac infiltrate consists initially of Ly6C^inter/hi^ populations within the alveolar and interstitial spaces. In contrast, by *day 4*, the Ly6C^inter/lo^ mono-mac events remain constant in number whereas the alveolar and interstitial Ly6C^hi^ populations disappear. Of note, there are initial reductions in CD11c^pos^CD11b^neg^F4/80^pos^ alveolar macrophage populations at 24 h (Fig. 8*C*). Interestingly, the number of these CD11c^pos^CD11b^neg^F4/80^pos^ cells increases threefold (compared with uninjured numbers) on *day 2* during the insurgence of these exudative mono-macs. As previously described, it is from *day 2* onward that injury resolves within this model (44), and coincident with this are reductions in lung neutrophils (likely through apoptosis).

**Fig. 9.**
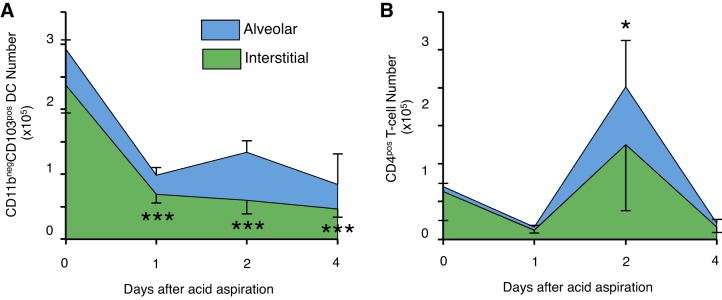
Changes in compartmental CD11b^neg^CD103^pos^ conventional dendritic cells (*A*) and CD4^pos^ T cells (*B*) during lung injury. There are reductions in dendritic cells possibly suggesting emigration to nearby lymph nodes. There is a significant increase in alveolar CD4^pos^ T cells on *day 2*. Data are means ± SD with *N* = 3–5/time point; *P < 0.05 ***P < 0.001 vs. *day 0* (analysis performed within compartments).

To further characterize changes in leukocyte populations within the alveolar and interstitial compartment during resolution, we focused on CD11b^neg^CD103^pos^ dendritic and CD4^pos^ T cell populations (Fig. 9). As expected, in the uninjured lung there are significant proportions of interstitial CD4^pos^ T cells. During acid-induced lung injury there were significant reductions in CD103^pos^ dendritic cell populations from *day 1* of injury, which persisted throughout the injury period studied. Additionally, there were significant increases in alveolar CD4^pos^ T cell populations on *day 2* during mono-mac migration into the lung.

## DISCUSSION

In this study we developed and validated a novel in vivo labeling method that enables an in-depth analysis of leukocyte location and phenotypes within the alveolar, interstitial, and vascular compartments of the mouse lung by flow cytometry. The protocols described here enable the positive identification of leukocytes within the lung vascular and alveolar compartments, and through a negative gating strategy the identification of the interstitial compartment. Combining this in vivo labeling strategy with in vitro use of a panel of multiple leukocyte surface markers, we achieved robust differentiation and identification of various leukocyte populations across three lung compartments in both the uninjured and injured mice. We showed that CD11c and CD11b are very useful global discriminators of myeloid cell populations in various compartments of the mouse lung. We confirmed clear separation between the alveolar and interstitial compartments through establishing distinct locations of resident alveolar macrophages (CD11c^pos^CD11b^neg^MHCII^neg^CD103^neg^ F4/80^pos^SiglecF^pos^ events) and two interstitial dendritic cells (CD11c^pos^CD11b^neg^ MHCII^pos^CD103^pos^ and CD11c^pos^CD11b^pos^MHCII^pos^CD103^neg^ events). An important distinction was found between the interstitial and vascular CD11c^neg^CD11b^pos^Ly6G^lo^Ly6C^lo^ monocyte populations, with the former showing significantly higher MHCII expression, similar to dendritic cells. On application to a clinically relevant mouse model of lung injury with substantive alveolar-capillary disruption, we presented the validity of this protocol and described, for the first time, the dynamics of leukocyte populations within the various lung compartments during inflammatory and resolution phases of acute lung injury.

Current methods for compartmental analyses of lungs have critical limitations for determination of location and phenotype of leukocytes. Despite recent advances in lung biology research, there has been no study to accurately and reliably address the precise numbers and phenotypes of leukocytes within each lung compartment (5, 24, 33, 58), mainly because of the unique morphology of the lung with very thin alveolar-capillary structures, which significantly limits the application of traditional histological techniques. In particular, investigation into the interstitial compartment of the lung has long proven elusive. Johnston et al. (24) performed cardiac perfusion and lavage procedures to determine which cells were present within the lung interstitium. However, we have demonstrated that standard lung lavage does not retrieve the majority of cells from the alveolar space. More aggressive lavage procedures (e.g., using EDTA) would damage the lung parenchyma, leading to contamination of cells from the interstitial and vascular spaces. Importantly, after the onset of lung injury the recoverability of alveolar macrophages would substantively worsen as a consequence of increased activation and adherence, a concept called “the macrophage disappearance reaction” (7, 20, 34). Our results have also shown that lung perfusion does not remove all of neutrophils from the pulmonary vasculature, consistent with our previous IPL study on monocytes (56), and those marginated leukocytes adhered relatively firmly to the lung endothelium would play a substantial role in the pathophysiology of lung injury (2, 39–41, 56, 57). In addition, lung perfusion following a period of zero pulmonary flow (during surgical preparation) may produce activation of endothelial cells (9, 10), stimulating production of reactive oxygen species (50) and promoting neutrophil adherence/recruitment to the lung (36), which may further reduce the recovery of vascular leukocytes.

Recent studies have also provided panels of markers to distinguish between various myeloid cells within the lung (1, 5, 24, 33, 58). However, such panels do not distinguish the anatomical localization of leukocytes and do not account for the potential changes in phenotypic markers during various stages of lung injury. In particular, macrophages show significant plasticity in their cell surface markers during injury and resolution (1). Our in vivo labeling method should supplement these myeloid marker panels but, importantly, is not restrictive to myeloid cells and has the added benefit to also enable the localization of other immune cells, e.g., T cells. Indeed, it is also feasible to perform in vivo labeling with alternative antibodies, or antibody cocktails.

Bedoret et al. (6) characterized resident alveolar macrophages, interstitial macrophages, and dendritic cells using immunohistochemistry and extensive lung lavage and showed that alveolar macrophages were CD11c^pos^F4/80^pos^MHCII^neg^, interstitial dendritic cells were CD11c^pos^F4/80^neg^MHCII^pos^, and interstitial macrophages were CD11c^neg^F4/80^pos^MHCII^pos^. The nomenclature of monocytes vs. macrophages remains very confusing within the literature. In contrast to the article by Bedoret, here we have defined mature macrophages as those cells that exhibit clear CD11c expression within the tissues. Such blood-borne Ly6C^hi^ monocyte-derived cells can differentiate either into macrophages (or mono-mac) or dendritic cells (mono-DC) (54, 60). Interestingly, we have found that these resident interstitial monocytes, unlike vascular monocytes, show a significant increase in CD86 expression within a model of lung ischemia-reperfusion (unpublished results). Taken together with the high expression of MHCII, this suggests a potential important immune modulatory role for these previously “invisible” cells, in particular, a link to adaptive T cell responses.

It is important to consider the potential confounding factors that would impede the ideal separation of lung compartments in our methodology. The use of negative selection would theoretically more or less “overestimate” the interstitial compartment as 100% labeling by intratracheal and intravascular antibody, which would not be possible, e.g., owing to some nonperfused/occluded vessels in the pulmonary vasculature. However, Reutershan et al. (47) intravenously administered a monoclonal antibody (against TER-119 on erythrocytes) and showed near total labeling of all erythrocytes in the pulmonary microvasculature of C57BL/6 mice. Similarly, our intratracheal antibody showed labeling of over 98% of resident alveolar macrophages within the alveolar space with less than 2% unstained.

On the other hand, if intravenous or intratracheal antibodies inadvertently label cells in other compartments either in vivo or during sample processing, e.g., unbound excess antibodies left within the lung samples bind to cells in other compartments, it would “underestimate” the interstitial compartment. However, such “leaks” of in vivo administered antibodies to other lung compartments appeared minimal in this study, because double-positive events for intratracheal and intravascular antibodies (expected to occur if significant bidirectional leaks take place) were always less than 0.5% of total events, and this proportion remained unchanged even in acid-injured mice with significant alveolar-capillary barrier disruption. Furthermore, the majority of these double-positive events were secondary to autofluorescence of resident alveolar macrophages.

Several precautions incorporated into our in vivo labeling protocol may have helped to minimize such leak. Firstly, intravenous CD45 antibody was injected and allowed to circulate for only 5 min. In our previous study using the same acid model, when fluorescence-labeled albumin (with a molecular weight of 66.5 kDa, compared with 160 kDa for anti-CD45 IgG) was injected intravenously to measure permeability index, we found that at least 30 min is required to detect measurable amounts of albumin in lung lavage fluid (43). Thus, within the time frame used in this study, it is unlikely that sufficient amounts of intravenous antibody are translocated into other lung compartments despite a substantively disrupted alveolar capillary barrier. Secondly, mice were exsanguinated (bled from inferior vena cava under systemic anticoagulation) and their lungs were inflated for 5 min with a positive alveolar pressure (with the intratracheal anti-CD45 antibody solution), which enables maximal drainage of pulmonary blood (and excess intravenous antibody). Further removal of excess intravascular antibody can be obtained by flushing the pulmonary vasculature, although this was not performed in this study to maintain lung architecture as intact as possible. Thirdly, to minimize the binding of any remaining unbound intratracheal antibody (left within the alveoli and bronchial tree), steps were implemented, including homogenization of the lung in the largest possible fluid volume followed by immediate dilution into 20 ml of ice-cold flow cytometry wash buffer. Drainage of excess antibody out of the tracheobronchial tree was not performed, given the potential for significant loss of cells from the alveolar compartment. An alternative method would be to homogenize the lung in excess nonconjugated anti-CD45 antibody to saturate the CD45 epitope, preventing further conjugated antibody binding.

The compartmental analysis enables a simple yet dynamic insight into the kinetics of leukocyte migration within the lung during inflammatory pathology. Acid-induced lung injury induced gradual increases in CD11b-positive events mainly on *day 2*, consistent with our previous work (44). In addition to neutrophils, we found a dual neutrophil and monocyte recruitment into the lung interstitium at this time point, as has been suggested in an endotoxin model of lung injury (17). There are also significant increases in Ly6c^hi^ monocytes within blood at this stage (data not shown) coinciding with their substantial increase in the interstitial and alveolar compartments. Interestingly, on *day 4*, this infiltrating Ly6c^hi^ monocyte population adopts a Ly6c^lo^ phenotype within the interstitium whereas it adopts a Ly6c^inter^ phenotype within the alveolar space. Hence, these data are consistent with the notion that exudative Ly6c^hi^ mono-macs could serve to replenish resident populations (dendritic cells and resident alveolar macrophages are, respectively, Ly6c^lo^ and Ly6c^inter^) within the lung after injury (29, 32). This is also suggested by the increase in numbers of resident alveolar macrophages on *day 2* after their initial reduction on *day 1*. Landsman et al. (29) showed that Ly6c^hi^ monocytes could give rise to alveolar macrophages after having adopted a Ly6c^lo^ phenotype, potentially explaining the sustained alveolar presence of Ly6c^lo^ monocytes.

Janssen et al. (23) compared dynamics of resident and recruited macrophages in model of endotoxin-induced lung injury. To place this into context with our study, which shows an increase in the CD45^pos^CD11c^pos^CD11b^neg^F4/80^pos^ alveolar macrophage pool, the Janssen study also showed a small increase in resident alveolar macrophages after a dramatic increase in recruited macrophages. However, their study only analyzed cells within bronchoalveolar lavage and, therefore, should have substantially underestimated the precise numbers of resident and recruited macrophages within the alveolar compartment. Furthermore, it is important to consider the relative efficacies of retrieval by lung lavage between various immune cell types (e.g., resident vs. monocyte-derived exudative macrophages; newly recruited vs. apoptotic exudative macrophages) and between various phases of injury (e.g., inflammatory vs. resolving phases). Our protocol of in vivo labeling removes many of these potential sources of error intrinsic to methods reliant on lung lavage.

On *day 4*, our data show reductions in Ly6c^hi^ monocytes with concomitant increases in Ly6c^lo/inter^ monocytes, consistent with evidence in other models of inflammatory resolution of a phenotypic switch from Ly6C^hi^ to Ly6C^lo^ (11, 46). During sterile injury, this switch from inflammatory Ly6C^hi^ to resolving Ly6C^lo^ mono-mac phenotypes would imply an increased phagocytosis potential and hence could explain the reductions in neutrophils (through apoptosis and efferocytosis) within the alveolar space (11). Plantinga et al. (45) showed that monocytes rapidly downregulate Ly6C on differentiation to mono-DCs within the lung and suggest this to be a highly specific effector of T cell responses. Simultaneously with this transition in mono-mac Ly6C phenotype, we also found increases in CD4^pos^ T cells within the interstitium at *day 2*. Furthermore, circulating Ly6C^hi^ monocytes have been shown to differentiate into fibrocytes under the influence of CD4^pos^ T cells (35), and Ly6c^hi^ monocytes have been implicated in driving fibrosis within a bleomycin-induced lung injury model (21). Interestingly, our model of acid aspiration produces significant fibrosis and collagen deposition from *day 2* (44). Whether the highly immunomodulatory subset of T cells (CD4^pos^CD25^pos^FoxP3^pos^) affects such changes in mono-mac Ly6C phenotype and ultimately resolution of inflammation (12, 13) has yet to be determined.

The complex interactions between innate and adaptive immune responses facilitated by dendritic cells have been of considerable interest in numerous mucosal surfaces including the gut and skin (30, 54). Indeed, CD11b^neg^CD103^pos^ intraepithelial cDCs bind to epithelial cells using the α_E_β7 integrin CD103, the only known ligand for the epithelial integrin E-cadherin (31, 53). Their early loss during acid-induced lung injury may reflect the significant epithelial injury induced within this model (44). Indeed, these CD103^pos^ cDCs have been shown in the gut epithelium to initiate adaptive immune responses in local lymph nodes (49) and within the lung to transport apoptotic cell-associated antigens to draining lymph nodes (16). A limitation of our study was the restriction to a seven-channel flow cytometer, and only five channels after dual intravenous and intratracheal labeling. Additional channels to differentiate CD11b^pos^CD103^neg^ dendritic cell populations from infiltrating CD11b^pos^ mono-DCs by using antibodies against CD68 or MAR-1 would have further increased the depth of our analysis (45). More detailed compartmental analysis of the lungs could potentially yield invaluable insights into the roles of such leukocyte interactions and transitions during inflammation and its resolution.

In conclusion, we present an in vivo antibody-labeling methodology to analyze the subsets of myeloid cells within the three compartments of the lung. This protocol takes full advantage of the multiparameter analysis capabilities of flow cytometry and enables robust discrimination of the location and phenotype of leukocytes within healthy and injured mouse lungs. Combining this protocol with more recent myeloid cell panels and technologies such as conditional/inducible gene modification would substantively facilitate investigations into transcompartmental trafficking of leukocytes within the lungs. We propose application of this simple in vivo lung compartmental analysis to other lung biology research to discover novel insights into the roles of leukocytes within the lung.

## DISCLOSURES

No conflicts of interest, financial or otherwise, are declared by the author(s).

## AUTHOR CONTRIBUTIONS

B.V.P., K.C.T., M.R.W., and M.T. conception and design of research; B.V.P. and K.C.T. performed experiments; B.V.P. and K.C.T. analyzed data; B.V.P., K.C.T., K.P.O., and M.T. interpreted results of experiments; B.V.P. prepared figures; B.V.P. drafted manuscript; B.V.P., K.C.T., M.R.W., K.P.O., and M.T. edited and revised manuscript; B.V.P. and M.T. approved final version of manuscript.
